# MYC Regulates α6 Integrin Subunit Expression and Splicing Under Its Pro-Proliferative ITGA6A Form in Colorectal Cancer Cells

**DOI:** 10.3390/cancers10020042

**Published:** 2018-02-03

**Authors:** Jean-François Groulx, Salah Boudjadi, Jean-François Beaulieu

**Affiliations:** 1 Laboratory of Intestinal Physiopathology, Department of Anatomy and Cell Biology, Faculty of Medicine and Health Sciences, Université de Sherbrooke, Sherbrooke, QC J1H 5N4, Canada; jean-francois.groulx@usherbrooke.ca (J.-F.G.); salah.boudjadi@usherbrooke.ca (S.B.); 2 Laboratory of Pathology, Cancer Molecular Pathology Section, National Cancer Institute, Bethesda, MD 20892, USA

**Keywords:** colorectal cancer, MYC, integrin α6β4, ITGA6, alternative splicing, ESRP2

## Abstract

The α6 integrin subunit (*ITGA6*) pre-mRNA undergoes alternative splicing to form two splicing variants, named ITGA6A and ITGA6B. In primary human colorectal cancer cells, the levels of both ITGA6 and β4 integrin subunit (ITGB4) subunits of the α6β4 integrin are increased. We previously found that the upregulation of ITGA6 is a direct consequence of the increase of the pro-proliferative ITGA6A variant. However, the mechanisms that control ITGA6 expression and splicing into the ITGA6A variant over ITGA6B in colorectal cancer cells remain poorly understood. Here, we show that the promoter activity of the *ITGA6* gene is regulated by MYC. Pharmacological inhibition of MYC activity with the MYC inhibitor (MYCi) 10058-F4 or knockdown of MYC expression by short hairpin RNA (shRNA) both lead to a decrease in ITGA6 and ITGA6A levels in colorectal cancer cells, while overexpression of MYC enhances *ITGA6* promoter activity. We also found that MYC inhibition decreases the epithelial splicing regulatory protein 2 (ESRP2) splicing factor at both the mRNA and protein levels. Chromatin immunoprecipitation revealed that the proximal promoter sequences of *ITGA6* and *ESRP2* were occupied by MYC and actively transcribed in colorectal cancer cells. Furthermore, expression studies in primary colorectal tumors and corresponding resection margins confirmed that the up-regulation of the *ITGA6A* subunit can be correlated with the increase in *MYC* and *ESRP2*. Taken together, our results demonstrate that the proto-oncogene MYC can regulate the promoter activation and splicing of the *ITGA6* integrin gene through ESRP2 to favor the production of the pro-proliferative ITGA6A variant in colorectal cancer cells.

## 1. Introduction

Integrins are the principal transmembrane receptors responsible for cell–extracellular matrix interactions. They are formed by the non-covalent association of αβ dimers. To date, 18 α and 8 β subunits have been found, leading to the formation of 24 distinct integrins [1]. However, alternative splicing and post-translation modifications increase the number of integrins with distinct functions in humans [2]. While integrin receptors lack intrinsic kinase domains, their cytoplasmic domains can associate with a panel of intracellular adaptors and kinase proteins for the regulation of a variety of cell signaling pathways and cell functions involved in events associated with cancer progression including migration, invasion, proliferation, differentiation and survival [3,4,5]. Several integrins have been reported to be overexpressed in malignant cells [6,7]. Among them, the expression of α6β4 has been particularly well documented in carcinomas [8]. The α6β4 integrin is a unique integrin, with a β4 subunit (ITGB4) displaying an elaborate cytoplasmic domain of more than 1000 amino acids and the α6 subunit (ITGA6) as its only possible α subunit partner [9]. In normal epithelia, this laminin receptor promotes stable epithelial cell adhesion through its participation with hemidesmosomes, complex adherent junctional structures that connect intermediate filaments to the basement membrane [10]. However, in carcinomas, the α6β4 integrin is released from hemidesmosomes and becomes associated with actin-related motile structures involved in the activation of multiple signal transduction cascades susceptible to contributing to tumor progression in terms of enhanced proliferation, cell survival, invasion and metastasis [8,11].

In agreement with other studies [8], our laboratory has reported that both subunits of the α6β4 integrin are upregulated in primary tumors of patients with colorectal cancer (CRC) [12,13]. It is noteworthy that in various tissues, the β4 subunit is found to be susceptible to proteolysis [14,15] while the *ITGA6* subunit can undergo alternative splicing of its exon 25, leading to the formation of two distinct variants, identified as ITGA6A and ITGA6B [16], suggesting a potential variation in downstream signalling depending on the α6β4 form. In the intestine, ITGB4 and ITGA6 were initially found to be ubiquitously expressed in all epithelial cells of the crypt-villus axis [17,18]. Later on, it was found that, in contrast to mature cells which express an intact β4 subunit, proliferative/immature cells of both the small intestinal and colonic glands express a proteolytically processed β4 subunit that lacks the C-terminal segment of the cytoplasmic domain leading to an α6β4ctd integrin not functional for adhesion to laminin [13,19]. The α6A/B variants were also found to be differentially expressed along the intestinal and colonic crypt axes, ITGA6A expression is restricted to the proliferative and undifferentiated cells of the crypts, whereas ITGA6B was found in the quiescent and differentiated cells of the small intestinal villus and colonic surface epithelia [12,20] consistent with its anti-proliferative influence on CRC cell proliferation [12]. Incidentally, in CRC cells, the predominant α6β4 form identified appears to be α6Aβ4ctd+, a hybrid heterodimer not found in normal intestinal or colonic epithelial cells [21]. Further studies led to the identification of ITGA6A as a pro-proliferative β4 integrin partner regulating the Wnt/β-catenin pathway and tumorigenesis in CRC [22]. Importantly, we also found that the overexpression of ITGA6 is a direct consequence of a net increase in the expression of the oncogenic ITGA6A variant [22].

These findings suggest the occurrence of hypothetical common mechanisms promoting *ITGA6* expression and preferential splicing into the *ITGA6A* form in CRC cells but at this time, the regulation of *ITGA6* expression and splicing in CRC remains poorly understood. Indeed, on one hand, the alternative splicing of *ITGA6* has been well documented over the last few years, being potentially regulated by epithelial splicing regulatory protein 1 and 2 (ESRP1 and ESRP2) [23,24,25,26], RNA Binding Motif Protein 47 (RBM47) [24], the RNA-binding protein Muscleblind (MBNL1) and the RNA-binding protein FOX2 homologue (RBFOX2) [27] as well as the polypyrimidine tract-binding protein 1 (Ptbp1) [28], but the potential influence of these factors on *ITGA6* splicing has not been studied in the context of colorectal cancer cells. On the other hand, information on the transcriptional regulation of *ITGA6* remains sparse as consensus binding sites for specificity protein 1 (SP1), nuclear factor-κB ( NF-κB), activator protein 1 (AP1) and MYC were identified in the *ITGA6* promoter two decades ago [29] but only the SP1/SP3 sites appear to have been confirmed by chromatin immunoprecipitation (ChIP) analysis [30].

Considering that the proto-oncogene MYC controls the expression of a large array of genes [31] including those encoding various integrin subunits [13,32,33], is upregulated in up to 70% of CRC [34,35] and has been shown to be a modulator of various slicing regulators in cancer cells [36], we hypothesized that MYC could be involved in the up-regulation of ITGA6 expression and splicing in CRC. In this study, we confirmed this hypothesis by highlighting a novel mechanism by which MYC can directly control both *ITGA6* and *ESRP2* promoter activities in CRC cells, leading to the overexpression of the pro-proliferative ITGA6A splice variant.

## 2. Results

### 2.1. MYC and ITGA6A Expression Correlate in CRC Cells

**Figure 1:**
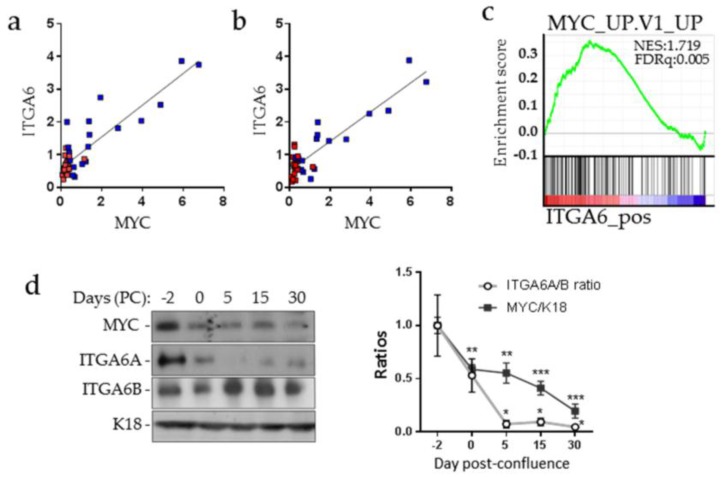
Integrin α6A (ITGA6A) and MYC expression correlation in colorectal cancer cells. Correlation between *ITGA6* (**a**) and *ITGA6A* (**b**) with *MYC* transcript expression was assessed in 20 primary colorectal cancer tumors (blue squares) and their resection margins (red squares). Pearson r = 0.8802; *p* ≤ 0.0001 (*ITGA6A* vs. *MYC*); Pearson r = 0.8933; *p* ≤ 0.0001 (*ITGA6* vs. *MYC*).(**c**) Positive correlation observed on a gene set enrichment analysis GSEA plot of enrichment of *MYC* upregulated genes in high *ITGA6* expression specimens from the human colorectal cancer data set (GSE35896) using C2 MSigDB database.(**d**) Representative Western blot analysis of ITGA6A, ITGA6B and MYC expression in Caco-2/15 cells in sub-confluent (−2 days), confluent (0 day) and post-confluent (5–30 days) monolayers and quantification of ITAG6A/ITGA6B and MYC/K18 ratios through the confluence. Cytokeratin 18 (K18) was used as a loading control. Statistically significantly different from -2 days post-confluence: * *p* ≤ 0.05; ** *p* ≤ 0.01; *** *p* ≤ 0.001. Pearson r = 0.9005 *p* ≤ 0.05 for ITGA6A/ITGA6B ratios vs. MYC/K18.

The possible involvement of MYC in ITGA6A expression was first tested by qPCR on a panel of human CRC samples. As expected from previous studies [22,32], *MYC, ITGA6* and *ITGA6A* mRNAs were found to be significantly increased in CRC relative to their matched resection margins and a close correlation between both *ITGA6* and *MYC* and *ITGA6A* and *MYC* expression was observed (Figure 1a,b), consistent with the gene expression profiling of *ITGA6* in public data sets of gene expression of colorectal cancer (GSE35896) revealing a positive correlation between *ITGA6* expression and *MYC*-induced genes (Figure 1c). It is noteworthy that *ITGA6B* expression was not modulated in tumors relative to their corresponding resection margins [22]. In Caco-2/15 cells, which undergo a spontaneous switch from the ITGA6A to ITGA6B form after confluence [20], we also observed a significant correlation with the reduction of MYC during the same period (Figure 1d) supporting a possible link between MYC and ITGA6A expression.

### 2.2. MYC Regulates ITGA6A Expression in CRC Cells

**Figure 2:**
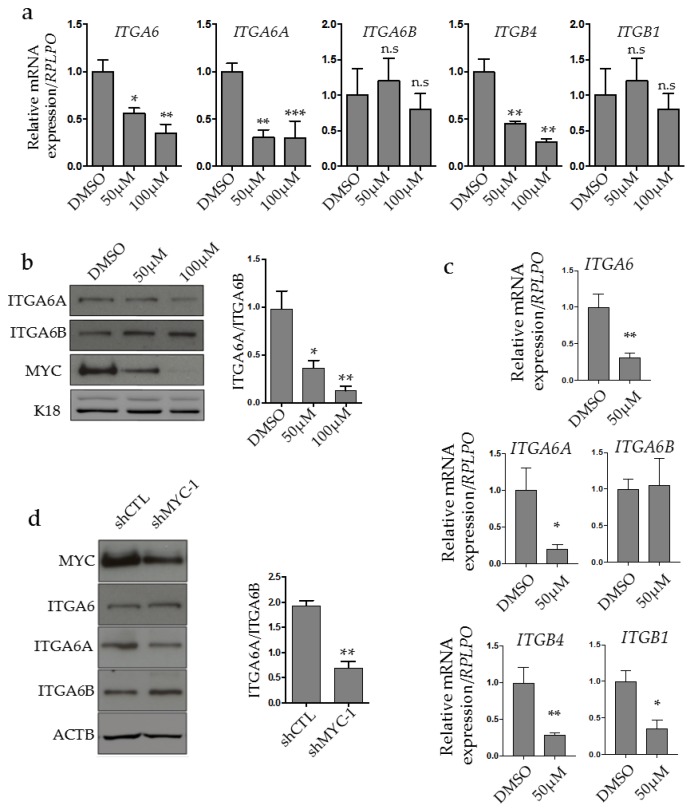
MYC regulates ITGA6 expression and splicing. (**a**) Quantitative RT-PCR analysis of *ITGA6, ITGA6A, ITGA6B, ITGB4* and *ITGB1* integrin subunits in T84 CRC cells treated with the MYC inhibitor at 50 and 100 μM for 48 h. * to ***: statistically significantly different (*p* ≤ 0.05 to 0.001) from dimethyl sulfoxide (DMSO) control. (**b**) Representative immunoblot and densitometric analyses of ITGA6A, ITGA6B and MYC expression in CRC cells treated with the MYC inhibitor at 50 and 100 μM for 48 h. Cytokeratin 18 (K18) was used as loading control. * to **: statistically significantly different *p* ≤ 0.05 to *p* ≤ 0.01 from DMSO control. (**c**) qPCR analysis of ITGA6, ITGA6A, ITGA6B, ITGB4 and ITGB1 in normal human intestinal epithelial cells (HIEC) treated with MYC inhibitor at 50 μM for 48 h. * to **: statistically significantly different (*p* ≤ 0.05 to *p* ≤ 0.01) from DMSO control. (**d**) Representative immunoblot and densitometric analysis of ITGA6, ITGA6A, ITGA6B expression in MYC-knockdown T84 CRC cells. * to **: statistically significantly different *p* ≤ 0.05 to *p* ≤ 0.01 from DMSO control. β-actin (ACTB) was used as loading control. n.s: not significant.

The possibility that MYC regulates *ITGA6A* expression was first investigated using the pharmacological MYC inhibitor (MYCi) 10058-F4 on the T84 CRC cell line. Cells were treated with 50 and 100 µM for 48 h prior to mRNA and protein analysis. qPCR analysis revealed that MYC inhibition led to a significant decrease of *ITGA6* and *ITGA6A* at both concentrations compared to DMSO-treated cells (Figure 2a), whereas the level of *ITGA6B* remained the same. Interestingly, the *ITGB4* subunit exclusive partner *ITGA6* also decreased after MYCi treatment whereas the *ITGB1* level was not altered. At the protein level, a decrease of ITGA6A and an increase of ITGA6B were observed after MYCi treatment (Figure 2b). Of note, treatment of CRC cells with MYCi led to a significant dose-dependent reduction of MYC protein (Figure 2b), as previously reported [32]. We observed similar results in normal human intestinal epithelial cells (HIEC) treated under the same conditions with the MYCi, except that both ITGB1 and ITGB4 integrin subunits were downregulated (Figure 2c). The reduction of *ITGB1* in response to the MYCi in the normal intestinal HIEC cells is consistent with the fact that *ITGB1* contains the cognate CACGTG E-box in its promoter [37] while previous experiments have shown that MYC only plays a minor role on the ITGB1 promoter in colorectal cancer cells [32].

The influence of MYC on ITGA6A expression was also investigated by a short hairpin (sh)RNA approach testing a shRNA specific for MYC on ITGA6 protein expression by Western blot analysis. Partial downregulation of MYC with shMYC-1 had no significant effect on total ITGA6 levels but resulted in a clear switch from the ITGA6A to ITGA6B form (Figure 2d). Altogether, these results with MYCi and shRNA indicate that MYC can regulate *ITGA6* expression and splicing to favor expression of its pro-proliferative variant *ITGA6A*.

### 2.3. MYC Controls ESRP2 Expression to Regulate ITGA6 Splicing

**Figure 3:**
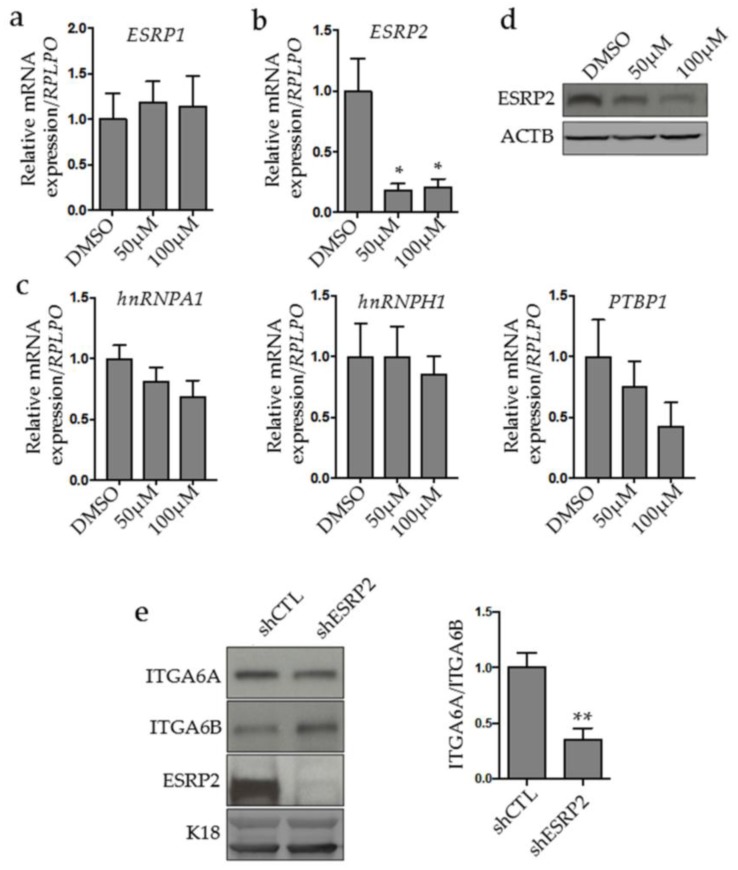
MYC regulates ITGA6 splicing through epithelial splicing regulatory protein 2 (ESRP2). Quantitative RT-PCR analysis of ESRP1 (**a**), ESRP2 (**b**) and heterogeneous nuclear RNA-binding protein H1 (hnRNPH1), hnRNPA1 and polypyrimidine tract-binding protein 1 (PTBP1) (**c**) mRNA expression in CRC T84 cells treated with the MYC inhibitor at 50 and 100 µM for 48 h. *: statistically significantly different (*p* ≤ 0.05) from DMSO control. Data are expressed normalized to *RPLPO* expression as a validated housekeeping gene. (**d**) Representative immunoblot analyses of ESPR2 protein level expression. β-actin (ACTB) was used as loading control. ESRP2 knockdown alters ITGA6 splice variant expression. (**e**) Representative immunoblot and densitometric analyses of ITGA6A, ITGA6B and ESRP2 in CRC cells knocked down for ESRP2. Cytokeratin 18 (K18) was used as loading control. **: statistically significantly different (*p* ≤ 0.01) from control sh (shCTL). 2.4. MYC Binds to ESRP2 and ITGA6 Promoters.

To gain insight into the nature of the mechanism underlying *ITGA6A* splicing regulation in CRC, we performed a screening of selected splicing factors that could be modulated by MYC to favor ITGA6A expression. Besides *ESRP1* and *ESRP2* that were selected on the basis of their documented effect on ITGA6 splicing in breast and renal neoplastic cells [25,26], respectively, we also tested the three MYC-controlled factors of the heterogeneous nuclear RNA-binding protein (hnRNP) family in cancer cells: *PTBP1, hnRNPA1* and *hnRNPH1* [38,39]. Interestingly, no change in *ESRP1* mRNA was observed by MYC inhibition in CRC cells (Figure 3a), while a drastic reduction of *ESRP2* was found at both the mRNA (Figure 3b) and protein levels (Figure 3c). This was clearly noticed even at the lowest concentration of the inhibitor, suggesting that MYC is an important regulator of ESRP2 expression. Analysis of the hnRNP family candidates *hnRNPA1*, *hnRNPH1* and *PTBP1* showed no change in their expression (Figure 3d). To further explore the possibility that ESRP2 is a splicing factor responsible for the MYC-dependent *ITGA6A* splicing in CRC, *ESRP2* expression was knocked down in CRC cells by shRNA and *ITGA6* splice variants were analyzed. Knockdown of ESRP2 resulted in a decrease in *ITGA6A* and an increase in *ITGA6B*, leading to a net decrease in the *ITGA6A/ITGA6B* ratio (Figure 3e). Together, these results demonstrated that ESRP2 is a key effector of MYC to regulate *ITGA6* splicing.

### 2.4. MYC Binds to ESRP2 and ITGA6 Promoters

**Figure 4:**
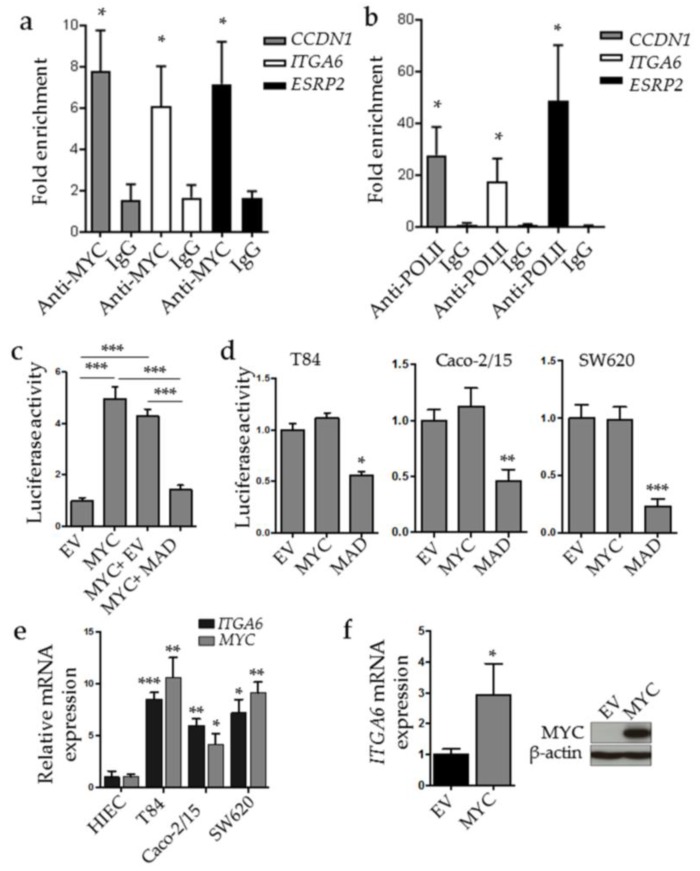
MYC binds to the *ITGA6* and *ESRP2* promoters. Chromatin immunoprecipitation (ChIP) assays were performed on T84 lysates using either anti-human c-Myc-specific antibodies (**a**) or anti Pol-II antibodies (**b**). Mock anti-IgG were used as negative control. The cyclin D1 *CCND1* promoter containing one complete E-box response element was used as a positive control. *: Statistically significantly different (*p* ≤ 0.05) from IgG. (**c**,**d**) Luciferase assay of the detection of *ITGA6* promoter activity. The *ITGA6* promoter coupled to Renilla luminescent reporter gene inserted into the pLightSwitch_Prom vector was transfected into HEK293T (**c**) or in CRC T84, Caco2-15 or SW680 (**d**) together with the empty vector (EV), a MYC expressing vector (MYC), the transcriptional dominant negative MADMYC (MAD) or both (MYC + MAD). Results showed the net Renilla/Firefly ratio relative to EV. * to ***: Statistically significantly different (*p* ≤ 0.05 to 0.001) from EV. (**e**) Quantitative RT-PCR analysis of *MYC* and *ITGA6* expression in HIEC, T84, Caco2-15 and SW620 cells. * to ***: Statistically significantly different (*p* ≤ 0.05 to 0.001) from HIEC cells. (**f**) Quantitative RT-PCR analysis of *ITGA6* expression in HIEC overexpressing MYC and representative immunoblot for MYC detection in the same cells. *: Statistically significantly different (*p* ≤ 0.05) from EV.

Incidentally, both *ESRP2* and *ITGA6* promoters contain one MYC binding sequence as determined by in silico analysis and a previous study [29], respectively, supporting a potential MYC regulation for both expression and splicing. To evaluate the capacity of MYC to bind to the proximal region of their promoters in the CRC context, we performed chromatin immunoprecipitation (ChIP) assays using T84 CRC cell lysates. Results demonstrated that MYC binds significantly to both *ESRP2* and *ITGA6* promoter sequences containing the MYC response elements, as well as to the cyclin D1 (*CCND1)* promoter as the positive control (Figure 4a). However, binding of MYC to a promoter does not indicate whether the promoter is activated or repressed. To test this possibility, we performed a ChIP assay to detect the presence of the RNA polymerase II (Pol-II) as an indicator of promoter activation. As expected, the positive control *CCND1* promoter sequence containing the E-box CACGTG showed a strong enrichment in Pol-II binding (Figure 4b). Our analysis also showed a significant enrichment of the same *ESRP2* and ITGA6 promoter regions with Pol-II. These results indicate that both the *ESRP2* and *ITGA6* promoters are positively controlled by MYC.

To further explore the impact of MYC on *ITGA6* integrin promoter activity, luciferase assays were performed using the *ITGA6* integrin promoter in various cell lines. In HEK293T cells, MYC overexpression led to a significant increase in *ITGA6* promoter activity compared with the empty vector (EV) control in these cells (Figure 4c). Furthermore, overexpression of a MADMYC construct (MAD), acting as a dominant negative of MYC [40], was sufficient to prevent the MYC activation of the *ITGA6* promoter (Figure 4c). However, MYC overexpression had no net effect on *ITGA6* promoter activation in CRC T84, Caco-2/15 and SW620 cells (Figure 4d) although *ITGA6*-luciferase activity can be inhibited by treatment with the MYCi as shown with SW620 cells (DMSO: 1.66 ± 0.21; MYCi: 0.99 ± 0.16, *p* < 0.01). This result suggests that MYC expression and activity is already at its maximum in those cells. Indeed, *MYC* mRNA expression levels were found to be significantly higher in these CRC cells compared to normal human intestinal epithelial crypt cells (HIEC) (Figure 4e) while in CRC cells, expression of the dominant negative MAD alone was sufficient to reduce the promoter activity of *ITGA6* (Figure 4d). Incidentally, transient expression of MYC in HIEC cells was found to be sufficient to stimulate *ITGA6* expression (Figure 4f). These results confirm the key role of MYC in the expression of both *IT6A6* and *ESRP2* by positively regulating their promoters.

### 2.5. ESRP2 Expression is Increased in CRC Cells in Cellulo and in Situ

**Figure 5:**
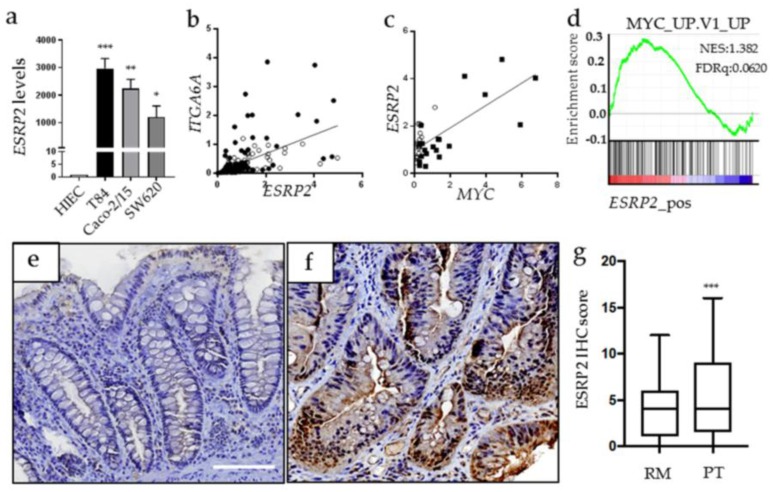
ESRP2 correlates with ITGA6A and MYC expression. (**a**) Evaluation of *ESRP2* mRNA expression in normal human intestinal epithelial cells (HIEC) and a panel of CRC T84, Caco-2/15 and SW620 cells. * to ***: Statistically different (*p* ≤0.05–0.001) from HIEC. (**b**) Correlation between *ITGA6A* and *ESRP2* transcript expression in a set of 84 primary CRC tumors and their corresponding resection margins (RM). Pearson r = 0.5161, *p* ≤0.0001. (**c**) Correlation between *MYC* and *ESRP2* transcript expression in a set of 20 primary CRC tumors and their resection margins (RM). Pearson r = 0.7243, *p* ≤ 0.0001. Open circle: RM; black square: CRC tumors. (**d**) GSEA plot of enrichment of MYC target genes of gene expression in high *ESRP2* expression from the human colorectal cancer data set (GSE35896) using C2 MSigDB database. (**e**,**f**) ESRP2 immunohistochemical staining on tissue microarrays (TMA) containing CRC primary tumors and corresponding resection margins for 49 patients. Representative staining from a patient where ESRP2 score was found to be higher in cancer (**f**) than in resection margin (**e**). Scale bar = 100 μm. (**g**) Distribution of immunostaining scores for ESRP2 in CRC primary tumors (PT) and corresponding RM. Boxes represent lower quartile, median, and upper quartile, whereas whiskers represents the lowest and highest immunostaining scores. ***: *p* <0.001, Wilcoxon test.

In comparison with MYC and ITGA6A expressions that are increased [12,32] and correlated in human colonic neoplastic cells (Figure 1a), little is known about ESRP2 expression in CRC cells. As expected from the above, ESRP2 was detected at high levels in all CRC cell lines by qPCR (Figure 5a). In CRC tumors and corresponding resection margins, ESRP2 expression was found to correlate with both ITGA6A (Figure 5b) and MYC (Figure 5c). Bioinformatic analysis of public data sets of human CRC also revealed a positive correlation between ESRP2 expression with MYC regulated genes. ESRP2 expression was also evaluated at the protein level on tissue microarrays prepared from 49 patients including tumors of all four stages and their corresponding resection margins. Only epithelial staining was considered. Expression of ESRP2 in CRC compared to corresponding resection margins was found to be increased in 30 patients (61.2%), similar in eight patients (16.3%) and reduced in 11 patients (22.5%). No significant relation with expression of ESRP2 and tumor stage was noted. Representative staining of increased ESRP2 expression in CRC vs its corresponding resection margin is illustrated (Figure 5e,f). Overall, the total ESRP2 score was found to be significantly higher in CRC than the resection margin (Figure 5g). Altogether, these observations emphasize the clinical relevance of the ESRP2-MYC-ITGA6A connection in CRC.

## 3. Discussion

In this study, we identified a mechanism by which MYC regulates ITGA6 expression and its splicing through an upregulation of ESRP2 to favor the ITGA6A splice variant, a pro-proliferative component of the α6β4 integrin in CRC cells. MYC is responsible for approximately 15% of total gene expression and is, thus, involved in the regulation of a broad range of cellular functions [31]. Therefore, it is not surprising that MYC has been found to regulate the spliceosome [41] leading to the formation of several pro-tumoral protein splice variants [42]. Here, we showed that MYC not only binds directly to ESRP2 and ITGA6 promoters, but also activates their transcription, demonstrated by ChIP and luciferase experiments. Our results clearly identified MYC as an activator of both genes’ expressions because the endogenous MYC activation of the ITGA6 promoter in cancer cells was inhibited by the expression of the repressor domain of the dominant negative MAD [40]. Upregulation of ITGA6 by MYC in CRC at the transcriptional level is consistent with the fact that MYC and ITGA6 have been found to be overexpressed separately in several types of cancers including breast [25,43,44] and prostate [45,46] cancers, liposarcoma [47,48] and glioblastoma [49,50].

To our knowledge, this is the first time that MYC has been identified as a direct regulator of ESRP2, as ChIP assays reveal that MYC binds to its promoter and that the gene is actively transcribed. As a consequence of being driven by MYC, we found that ESRP2 is overexpressed in CRC and strongly correlates with MYC levels, reinforcing their relationship. These observations are in agreement with the pro-oncogenic role suggested for ESRPs in CRC cells based on studies where high expression of ESPR1 stimulated anchorage-independent cell growth and ability to generate macrometastases in mouse livers [51]. In another study, ESRP1 was also found to enhance lung colonization of metastatic cancer cells, thus supporting a pro-metastatic function for ESRPs [52]. However, reduced expression of *ESRP1* and *ESRP2* transcripts has been reported by another group in CRC, in possible association with the epithelial–mesenchymal transition [53]. Indeed, ESRP1/2 can regulate the splicing of genes involved in epithelial–mesenchymal transition (EMT) and appears to need to be downregulated to achieve the EMT switch [23]. Unfortunately, ESRP2 expression was not analyzed at the protein level in the latter study [53]. Furthermore, MYC status was not determined in the tumors. Likewise, ESRPs role on tumor progression could be tissue and context specific.

Both ESRP1 and ESRP2 can regulate *ITGA6* splicing [23]. In breast cancer stem cells, ESRP1 is responsible for regulating ITGA6 splicing [25]. Herein, we found that MYC regulates ESRP2 expression, but not that of ESRP1, suggesting that in CRC, ESPR2 is the one responsible for the increase in *ITGA6A* splicing caused by MYC. This is also supported by the fact that, at the protein level, ESRP1 is overexpressed only in a subset of human CRC tissues (16%) [51], in comparison to 61% for ESRP2 found in our study. Nevertheless, it cannot be excluded at this time that ESRP1 may play a MYC-independent role on *ITGA6* splicing in normal tissues and some CRC tissues. The implication of other transcription factors on MYC-dependent *ITGA6A* expression in CRC cells cannot be ruled out either. In this context, it is interesting to note that ESRP2 expression was not found to be significantly modulated in differentiating Caco-2/15 cells. Indeed, MYC can modulate the expression of other splicing factors such as hnRNPA1, hnRNPA2B1 and PTBP1 [36,54] while as noted above, the splicing of *ITGA6* can be regulated by RBM47, MBNL1, RBFOX2 and PTBP1 in addition to ESRP [23,24,25,26,27,28]. The participation of additional splicing factors is thus likely. Considering that the spliceosome includes activator-splicing and repressor-splicing factors, it would be interesting to further decipher the mechanism that regulates ITGA6 splicing.

It is pertinent to note that one way by which ITGA6A promotes CRC cell proliferation is through the regulation of the Wnt/β-catenin pathway [22], for which MYC is a target gene, suggesting the existence of a feed forward loop that potentiates proliferation in CRC cells [37] while ITGA6B overexpression has previously been shown to negatively regulate MYC activity [12], supporting this interplay. MYC regulation of proliferation could therefore be, at least in part, driven by the upregulation of ITGA6A, which in turn depends on MYC for its expression and preferential splicing. It is interesting to note that the expression of α1β1, another pro-proliferative integrin, [55] and that of the β4 subunit, are also regulated by MYC in CRC cells [13,32]. In the normal colon, as for ITGA1 [32,55], ITGA6A is predominantly expressed in the proliferative region of the epithelium in the colonic crypts [12]. Incidentally, MYC expression is also restricted to the colonocytes located in the proliferative crypt compartment [32], suggesting that MYC regulates ITGA1 and ITGA6A expression under normal conditions as well. In HIEC cells, a normal human intestinal epithelial crypt cell line, downregulation of ITGA6 expression under its ITGA6A form in response to MYC pharmacological inhibition strengthens the possibility that MYC is also responsible for ITGA6A expression in crypt cells under normal conditions.

## 4. Materials and Methods

### 4.1. Human Colon Tissue Samples

Samples of CRC and paired resection margin tissues (at least 10 cm from the tumor) were obtained from patients undergoing surgical resection without prior neoadjuvant therapy. Tissues were obtained after patients’ written informed consent, according to protocols approved by the Institutional Human Subject Review Board of the Centre Hospitalier Universitaire de Sherbrooke. Diagnoses, staging and grading were performed by the pathologists of the Department of Pathology of the Centre Hospitalier Universitaire de Sherbrooke (Project ethic code #1991-17, 90-18, date of approval (27-08-2017).

### 4.2. Cell Culture, MYC and ESRP2 Stable Knockdown Cells and MYC Inhibition

All cell lines used in this study were obtained from the American Type Culture Collection (Manassas, VA, USA) or from the original investigators for Caco-2/15 [56] and HIEC cells. [57] Caco-2/15, SW620 and HEK293T cells were grown in Dulbecco’s modified Eagle’s medium (DMEM) (Life Technologies, Burlington, ON, Canada) supplemented with 10% fetal bovine serum, 2 mM GlutaMAX and 10 mM 4-(2-hydroxyethyl)-1-piperazineethanesulfonic acid (HEPES) at 37 °C and 5% CO_2_. T84 cells were grown in DMEM/F12 (Life Technologies) supplemented with 5% fetal bovine serum, 2.5 mM GlutaMAX, 15 mM HEPES and 0.5 mM pyruvate (Life Technologies). The normal proliferative human intestinal epithelial cells, HIEC, were grown as described previously. [58] All cell lines were authenticated using the Promega GenePrint 10 kit (Promega, Madison, WI, USA) for short tandem repeat (STR) DNA profiling at the McGill University and Génome Québec Innovation Centre (Montréal, QC, Canada) and routinely tested for mycoplasma contamination using the MycoSensor PCR kit (Agilent, Mississauga, ON, Canada). Generation of stable cell lines for MYC and ESRP2 knockdown cells were performed using lentiviruses prepared in HEK293T cells with MISSION^®^ shRNA plasmid pLKO.1-puro (Sigma-Aldrich, Oakville, ON, Canada) specific for each gene. For MYC knockdown, lentiviruses were prepared with shRNA plasmids targeting MYC sequences as previously described. [32] For ESRP2 knockdown, lentiviruses were prepared with shRNA plasmids containing the short hairpin RNA targeting human ESRP2 sequence: 5′-CCGGCGACATGTTCTTCTCCTTCTACTCGAGTAGAAGGAGAAGAACATGTCGTTTTTTG-3′. The shRNA non-targeting control used contained a sequence targeting the GFP sequence: 5′: CCGGGTGGGCATCAAAGACGTGTTTCTCGAGAAACACGTCTTTGATGCCCACTTTTTG-3′. For knockdown studies, T84 cells were plated at 60% confluence 24 h prior to infection with lentiviruses, infected for 48 h and then selected with puromycin (10μg/ml) (Qiagen, Mississauga, ON, Canada) for 10 days. Stable T84 cell lines were confirmed for knockdown by Western blot and kept for further experiments. For MYC inhibition experiments, T84 and HIEC cells were seeded at 2 × 10^5^ and 10 × 10^5^ cells/well, respectively, in 6-well plates. At 50% confluence, the cells were treated with 50 μM or 100 μM of the MYC inhibitor (MYCi) 10058-F4 (Sigma-Aldrich) for 48 h prior to analysis. DMSO treated cells were used as control. mRNA and total cellular proteins were extracted for subsequent analysis.

### 4.3. Protein Extraction and Western Blot

Cells were scraped in 1 Laemmli sample buffer under non-reducing conditions and processed for SDS-polyacrylamide gel electrophoresis using 8–12% gel as previously described [22,32] under reducing (for MYC and ESRP2) or non-reducing conditions (for ITGA6A and ITG6B). Proteins were transferred onto nitrocellulose membranes (GE Healthcare, Mississauga, ON, Canada) and blocked for 1 h in 5% milk. Primary antibodies were diluted in the blocking solution and incubated at 4 °C: anti-α6A 1/500 (1A10, Millipore, Etobicoke, ON, Canada), anti-α6B 1/500 (6B4, Millipore), anti-integrin α6 1/500 (GOH3), anti-ESRP2 1/1000 (GTX123665, Genetex, Irvine, CA, USA), anti-c-Myc 1/5000 (Y69, ab32072, Abcam, Toronto, ON, Canada), anti-β-actin 1/80,000 (C4, Millipore) and anti-cytokeratin 18 1/100,000 (CY-90, Sigma-Aldrich). Densitometric analysis was performed using ImageJ software (version 1.43u, National Institutes of Health, Bethesda, MD 20892, USA).

### 4.4. Transfections and Luciferase Assays

Colorectal cancer cells and HEK293T were transfected using Effectine reagent (Qiagen) according to the manufacturer’s instructions. Promoter activation reported by Renilla and luciferase activities was measured using the Dual Luciferase Reporter Assay System (Promega) as previously described. [13,32] The ITGA6 promoter coupled with the Renilla plasmid (Switch Gear Genomics, Menlo Park, CA, USA) was co-transfected with the empty pcDNA3.1 plasmid (Life Technologies), the wild type MYC (pcDNA-MYC) or the negative dominant MYC mutant (CVMT-MADMYC) plasmids as previously described, [13,32] according to each experimental condition. Briefly, HEK293T, T84, Caco-2/15 and SW620 cells were seeded at 5 × 10^4^ cells/well in 12-well plates. The next day or when the cells reached 40–60% confluence, the cells were transfected with 100 ng of the Renilla coupled ITGA6 promoter plasmid and/or 100 ng of pcDNA3.1, pcDNA-MYC, or CVMT-MADMYC plasmids. The internal transfection control, 2 ng of the luciferase coupled pGL3-control vector was co-transfected at the same time. Cells were kept under normal growth conditions after transfection and harvested for analysis 48-h post-transfection. Results are represented from three independent experiments, each performed in triplicate.

### 4.5. RNA Extraction, RT-PCR and Quantitative RT-PCR

RNA extraction from human colorectal cancer cell lines and HIEC were performed using RiboZol reagent (Amresco, Solon, OH, USA) using the manufacturer’s instructions, quality tested and reverse transcribed as previously described. [32] Complementary DNA amplified using primers specific for ITGA6, ITGA6A, ITGA6B, RPLP0, MYC, ITGB4 and ITGB1 have been described and validated previously. [13,22,32,59] The other primers used were: for ESRP1, forward 5′-AAGTCTGCGGACAGAGCATT-3′ and reverse 5′-AGCAGGAGCTGGAAATGTGT-3′; ESRP2, forward 5′-CCCCTGTTGCCTACTATCCA-3′ and reverse 5′-ACGCTGAGCAGATCCTTCAT-3′; hnRNPA1, forward 5′-AGGAGCCATTTTGAGCAATG-3′ and reverse 5′-TTAAGTGGGCACCTGGTCTT-3′; hnRNPH1, forward 5′-ATGGGCTTGTCAAACCAGTC-3′ and reverse 5′-CCTATGCAATGTTTGATTGAAAA-3′ and PTB1, forward 5′-AGGGCCTGACCAAGGACTAC-3′ and reverse 5′-TCTGGATCAGTGCCATCTTG-3′. Quantitative PCR monitored with Brilliant II SYBR QPCR Low ROX Master Mix (Agilent) was performed on a MX3000P Real-Time System (Stratagene, Mississauga, ON, Canada). Relative mRNA levels of each gene were normalized to RPLP0 expression [59] according to the Pfaffl method [60] following minimum information for publication of quantitative real-time PCR experiments guidelines [61]. All experiments were performed in triplicate of three biological replicates.

### 4.6. Chromatin Immunoprecipitation

ChIP assay was performed following the procedure previously described. [32] Genomic DNA was extracted from T84 cells previously crosslinked with 1% paraformaldehyde solution (Fisher Scientific, Ottawa, ON, Canada). DNA was then sonicated into 500 to 1000 bp fragments and precleared for 1 h with protein A agarose/salmon sperm DNA (Millipore). DNA was then incubated overnight at 4 °C with anti-c-Myc (9E10 ChIP grade, Abcam), anti-RNA-Pol-II (Millipore) or anti-IgG (Millipore) antibodies. The DNA-protein-antibody complexes were collected with agarose beads and washed. Immunoprecipitates were then eluted and DNA-protein crosslinks were reversed at 65 °C for 16 h. Samples were then treated with RNase A (Roche Diagnostics, Laval, QC, Canada) and proteinase K (Roche Diagnostics). After purification, DNA amplification was performed using real-time PCR targeting the promoter regions of ITGA6, ESRP2 and CCND1 containing E-box elements, using the following pairs of primers: ITGA6: forward 5′-AAGCGCTCCATAAACACCTG and reverse 5′-GTGCTACTCGGCAACCACAA-3′; ESRP2: forward 5′-TCCTGGGTTGAGTTCTGGC-3′ and reverse 5′-GGAGTCATGGCCGCAGA-3′; CCND1: forward 5′-GAAACTTGCACAGGGGTTGT-3′ and reverse 5′-GCCAAAGAATCTCAGCGACT-3′. As a negative control, a DNA segment without E-box elements was amplified and the primers were: forward 5′-GGAGGGAGAAACACCTATTTTA-3′ and reverse 5′-GGAACTTAAACTTCACCATGAG-3′. Results represent the mean of three independent experiments, with qPCR performed in triplicate. Enrichment of the *ITGA6* and *ESRP2* promoter regions containing the CACGTG E-box was calculated by real time PCR quantification relative to the amount of control chromatin without the response element for MYC. Enrichment for the CCND1 promoter containing one complete E-box response element was used as a positive control.

### 4.7. Tissue Microarray and ESRP2 Immunohistochemistry Staining

Tissue microarray (TMA) preparation and immunohistochemical staining were performed as previously described for ITGA1 and MYC expression. [32,62] The same protocol was used herein for ESRP2 using an antigen retrieval procedure based on citrate buffer [62] and the rabbit anti-ESRP2 antibody 1/200 (GTX123665, GeneTex) overnight followed by horseradish peroxidase (HRP)-conjugated goat anti-rabbit antibody 1/100 (GE Healthcare). Images were acquired using a FSX100 Olympus microscope (Olympus, Center Valley, PA, USA) at 20× magnification. ESRP2 staining intensity was graded as a negative/weak (score = 1), moderate (score = 2), strong (score = 3) or very strong (score = 4). ESRP2 staining percentage was graded as 0% to 25% (score = 1) 25% to 50% (score = 2), 50% to 75% (score = 3) or ≥75% (score = 4). Final immunostaining score (intensity X percentage of expression) ranged from 1 to 16. The expression index for each patient was defined as the difference between the expression score for the tumor and normal paired samples: no change (0), increased expression in cancer (positive index = 1), and decreased expression in cancer (negative index = 1).

### 4.8. Bioinformatic Analysis

Gene set enrichment analysis was performed using GSEA v2.0.14 software (http://www.broadinstitute.org/gsea/index.jsp) with 5000 gene set permutations using the metric Pearson and the collection c6.all. v6.0.symbols. GSE35896 corresponding to microarray data of gene expression data from 62 colorectal cancers was analyzed.

### 4.9. Statistical Analysis

Each experiment was performed on three biological triplicates, except where mentioned and representative results were shown. The data are expressed as the mean ± SEM, except where mentioned. Student *t*-test for unpaired samples, and one-way analysis of variance (ANOVA) followed by Bonferroni’s multiple comparison tests were used to test the significance of the data. *p* ≤ 0.05 was considered significant. All graphs and statistical calculations were performed using Prism 6.0 software (GraphPad Software, La Jolla, CA, USA).

## 5. Conclusions

In the present study, we show for the first time that the proto-oncogene MYC can regulate the promoter activation and splicing of the ITGA6 integrin gene through ESRP2 to favor the production of the pro-proliferative ITGA6A variant in colorectal cancer cells.
